# Mining Physicians’ Opinions on Social Media to Obtain Insights Into COVID-19: Mixed Methods Analysis

**DOI:** 10.2196/19276

**Published:** 2020-06-18

**Authors:** Abdullah Wahbeh, Tareq Nasralah, Mohammad Al-Ramahi, Omar El-Gayar

**Affiliations:** 1 Slippery Rock University of Pennsylvania Slippery Rock, PA United States; 2 Supply Chain and Information Management Group D’Amore-McKim School of Business Northeastern University Boston, MA United States; 3 Texas A&M University-San Antonio San Antonio, TX United States; 4 Dakota State University Madison, SD United States

**Keywords:** pandemic, coronavirus, COVID-19, social media, infodemiology, infoveillance, medical professionals, opinion analysis

## Abstract

**Background:**

The coronavirus disease (COVID-19) pandemic is considered to be the most daunting public health challenge in decades. With no effective treatments and with time needed to develop a vaccine, alternative approaches are being used to control this pandemic.

**Objective:**

The objective of this paper was to identify topics, opinions, and recommendations about the COVID-19 pandemic discussed by medical professionals on the Twitter social medial platform.

**Methods:**

Using a mixed methods approach blending the capabilities of social media analytics and qualitative analysis, we analyzed COVID-19–related tweets posted by medical professionals and examined their content. We used qualitative analysis to explore the collected data to identify relevant tweets and uncover important concepts about the pandemic using qualitative coding. Unsupervised and supervised machine learning techniques and text analysis were used to identify topics and opinions.

**Results:**

Data were collected from 119 medical professionals on Twitter about the coronavirus pandemic. A total of 10,096 English tweets were collected from the identified medical professionals between December 1, 2019 and April 1, 2020. We identified eight topics, namely actions and recommendations, fighting misinformation, information and knowledge, the health care system, symptoms and illness, immunity, testing, and infection and transmission. The tweets mainly focused on needed actions and recommendations (2827/10,096, 28%) to control the pandemic. Many tweets warned about misleading information (2019/10,096, 20%) that could lead to infection of more people with the virus. Other tweets discussed general knowledge and information (911/10,096, 9%) about the virus as well as concerns about the health care systems and workers (909/10,096, 9%). The remaining tweets discussed information about symptoms associated with COVID-19 (810/10,096, 8%), immunity (707/10,096, 7%), testing (605/10,096, 6%), and virus infection and transmission (503/10,096, 5%).

**Conclusions:**

Our findings indicate that Twitter and social media platforms can help identify important and useful knowledge shared by medical professionals during a pandemic.

## Introduction

The rapid spread of the novel coronavirus disease (COVID-19) has sparked alarm worldwide. The World Health Organization (WHO) has declared the rapidly spreading COVID-19 outbreak to be a pandemic, and countries around the world are grappling with surges in confirmed cases [1]. This outbreak has changed the lives of many people in many countries. With millions of people forced out of public spaces, many conversations about these phenomena now take place on social media [2].

However, the accuracy and credibility of this conversation is often concerning and challenging for public health officials [3], especially because the authors of this information are often unknown [4]. In addition, data available on public platforms such as Twitter provide unique insights that are challenging to identify due to data size, recentness, and geographic scale [5,6]. Misinformation is spreading rapidly as people struggle to understand how best to protect themselves and the people around them [7]. Therefore, it is important to ensure that people seek information from proper sources on social media platforms. Seeking information from these outlets ensures the flow of relevant, accurate, and high-quality information about the COVID-19 pandemic outbreak, which can help control the pandemic [8]. An example of a proper source is a medical professional.

Currently, social media platforms such as Twitter and Facebook are being used by medical professionals around the world and have become important players in the COVID-19 pandemic. These platforms are used by medical professionals to provide patient care and education [4], increase personal awareness of news and discoveries, and provide health information to the community [9]. Furthermore, these platforms are increasingly popular for sharing and debating scientific information [10,11]. For example, Glowacki et al [3] analyzed tweets about electronic cigarettes posted by physicians from two countries, the United Kingdom and the United States; they found that physicians discussed important topics such as the likelihood of electronic cigarette use among teenagers, Food and Drug Administration regulations on tobacco, measures of the sources of harm inherent to any kind of tobacco use, and references to a Harvard study on the effects of flavoring chemicals on the lungs. In addition, Alpert and Womble [12] addressed how physicians navigate Twitter and their challenges and benefits of using the platform. The results showed that physicians used Twitter for reach and presence, to express concerns and apprehension, for networking, news, and education, for patient engagement, and to advocate against misinformation. Finally, Chaudhry et al [13] addressed the extent to which oncologists used Twitter during annual meetings. The results showed that physicians mainly used Twitter to report clinical news from scientific sessions, discuss treatment issues, for promotion, and to provide social commentary.

In summary, prior research demonstrates the potential of mining social media to uncover useful information regarding a variety of health care–related issues. However, no study to date has examined communities of medical professionals on social media to identify themes and discussion topics about the COVID-19 pandemic. These posts can help identify topics that are important to the community and can serve as a gauge for measuring concerns about potential threats [3]. With the rapid outbreak of the COVID-19 pandemic and the global effort to fight it, this research aims to extract medical professionals’ insights about the coronavirus pandemic. From a practical perspective, the research identifies proactive actions, recommendations, and knowledge that can help control the pandemic.

## Methods

### Methodology

**Figure 1:**
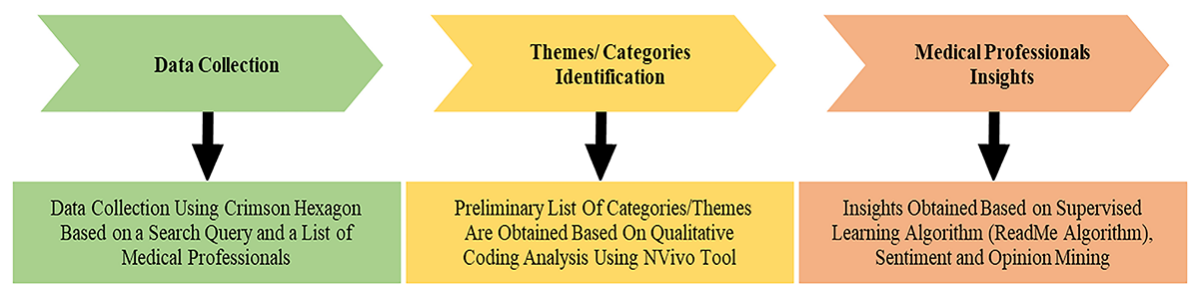
Research methodology.

To analyze posts by medical professionals on social media, we used a mixed methods approach blending the capabilities of a social media analytics tool, Crimson Hexagon, with the capabilities of a qualitative analysis tool, NVivo (QSR International), for data collection and analysis (Figure 1).

In general, the methodology started with data collection. The researchers agreed on a data range of interest, target social media platform, target users, keywords used to search for online posts, and restrictions to impose. Second, qualitative analysis was conducted using NVivo to explore the collected data to identify relevant tweets, infer prominent concepts, and then identify the main themes in the data. Qualitative analysis can be used to uncover important concepts and develop an understanding about a phenomenon [14]. A popular method for qualitative analysis is qualitative coding [15], which was adopted in this study. Qualitative coding is the process of assigning descriptive or inferential labels to chunks of data, which may assist concept development [16,17]. Third, a data analytics tool, Crimson Hexagon, was used for opinion analysis of the predefined categories. Crimson Hexagon, a social media analytics company that is now part of Brandwatch, employs unsupervised and supervised machine learning techniques and a text analysis model developed by Hopkins and King [18].

### Data Collection

**Figure 2:**
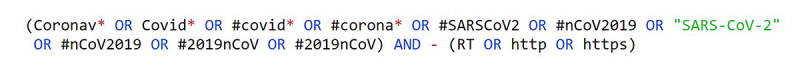
Search query used with Crimson Hexagon.

Our target social media platform for data collection was Twitter. Initially, we identified 119 medical professionals who were actively discussing the COVID-19 pandemic on Twitter. The medical professionals were identified by searching the Onalytica website, which specializes in providing influencer marketing software, and finding a list of top health care professionals ranked by influence score [19]. Also, we used the Johns Hopkins Coronavirus Resource Center [1], which provides a comprehensive COVID-19 case tracker as well as other useful information about COVID-19, including the Johns Hopkins COVID-19 Experts/Centers account on Twitter. The Twitter IDs of the medical professionals were used to identify the target users. Next, using Crimson Hexagon with the search query shown in Figure 2, we extracted all tweets for the identified medical professionals between December 1, 2019 and April 1, 2020. A total of 10,096 English tweets were collected. The key advantage of using a social media analytics platform such as Crimson Hexagon is that it provides access to the “Twitter firehose” (ie, every public tweet ever posted on Twitter in any language and from any geographic location that meets the search criteria).

### Data Analysis

For the data analysis, we started by identifying relevant tweets. To do this, a random subset of 250 tweets were analyzed by three researchers to determine which tweets were relevant to the COVID-19 pandemic. Three researchers independently labeled the tweets as relevant or not relevant. To ensure that the obtained results were reliable and consistent, we followed a case study protocol and established interrater reliability. We obtained a Fleiss κ value of 0.628, which is at the bottom of the range that reflects substantial agreement (0.61-0.80) and is just above the range that reflects moderate agreement (0.41-0.60) [20].

Next, we performed automatic coding of the relevant tweets using NVivo, a tool that helps organize and analyze a wide variety of data, including but not limited to documents, images, audio, video, and social medial content [21]. Automatic coding was used to assist the process of concept development related to the COVID-19 pandemic. The results of the automatic coding process are shown in Multimedia Appendix 1. Once the main codes and subcodes were identified, three researchers worked together via a combination of inductive and deductive thinking to identify conceptual categories [22]. The goal was to build a descriptive, multi-dimensional preliminary framework for later analysis. Based on the main code and subcode analysis, we were able to identify eight main categories/themes, namely information and knowledge, symptoms and illness, fighting misinformation, infection and transmission, testing, actions and recommendations, the health care system, and immunity.

We initially used Crimson Hexagon to define the categories from the qualitative analysis and the associated trained algorithm to explore the medical professional opinion surrounding the COVID-19 pandemic outbreak. Multimedia Appendix 1 describes each of the categories, lists the keywords delineating each of the categories, and provides a representative tweet for each. Using Multimedia Appendix 1 as a codebook, we manually labeled the categories and automatically distributed 10,096 tweets over 9 categories: the 8 categories from qualitative analysis and 1 additional category for irrelevant tweets. The training was an iterative process, ensuring that each category was clearly outlined by the examples. The number of coded tweets increased over several runs of the model as we reviewed the categories and coded more tweets.

## Results

### Tweet Distribution and Categories

**Figure 3:**
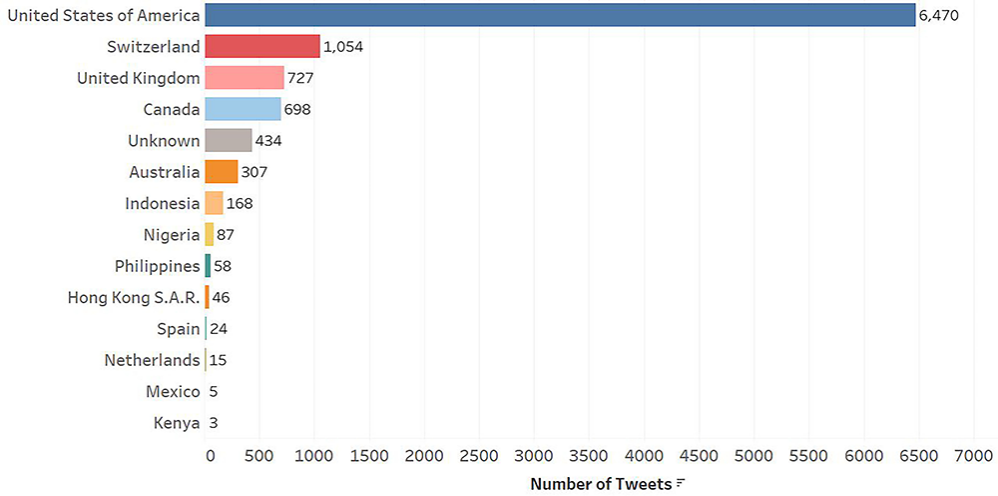
Distribution of tweets by country.

A total of 10,096 English tweets were collected between December 1, 2019 and April 1, 2020 from a total of 119 providers, of which 29 (24.4%) were female, 59 (49.6%) were male, and 31 (26.0%) were of unknown gender. The average number of tweets per provider was 84.8. The distribution of tweets per country is shown in Figure 3; the majority of tweets were from medical professionals located in the United States.

**Figure 4:**
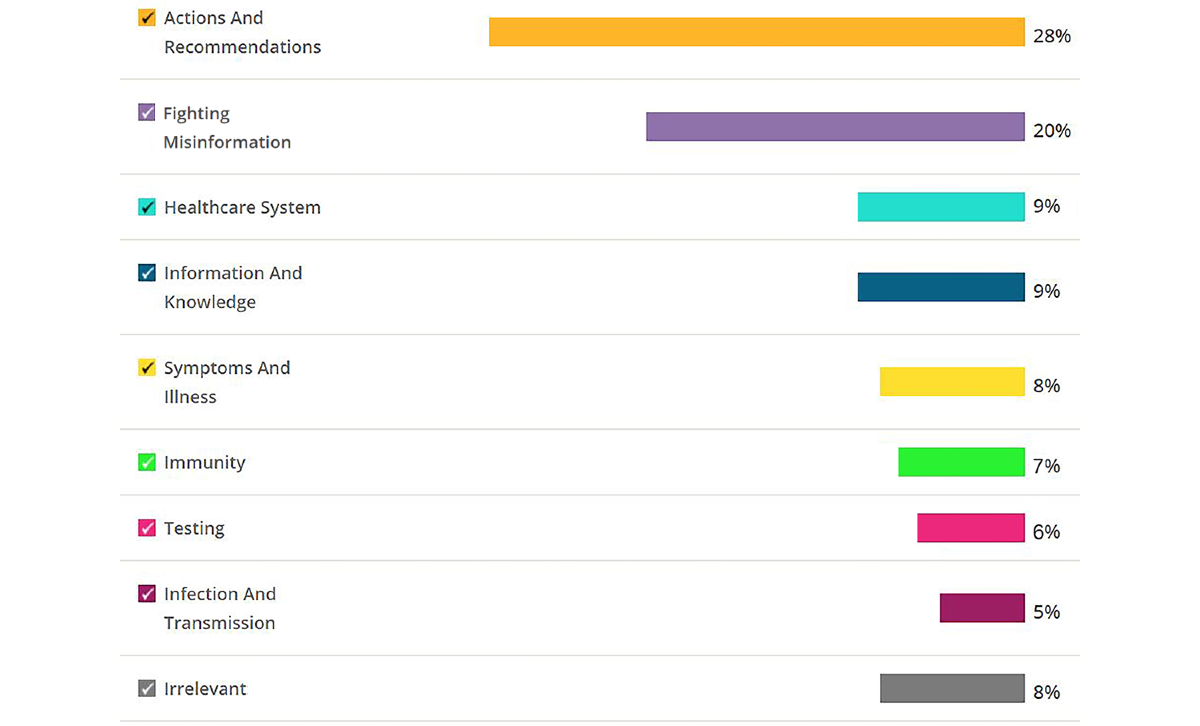
Percentages of tweets per category.

The distribution of the tweets over the categories identified using qualitative analysis is shown in Figure 4. Overall, the results demonstrate that relevant tweets account for 92% of the collected tweets, and irrelevant tweets account for 8%. Irrelevant tweets are tweets posted by medical professionals that do not discuss COVID-19. For example, tweets that reference websites links, such as “words have never been more well-spoken.
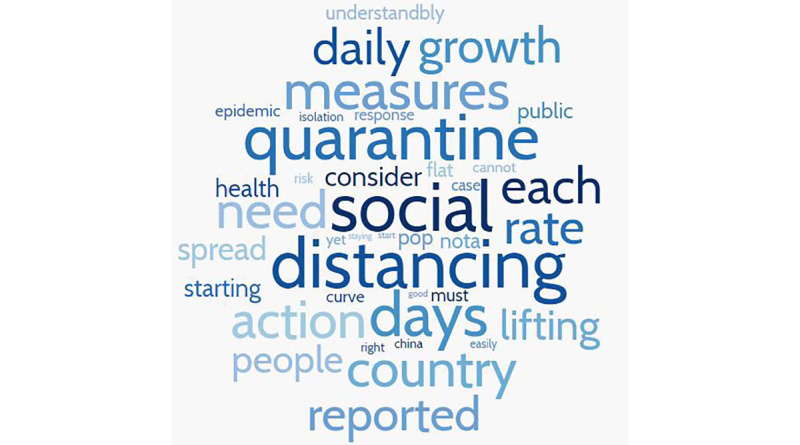
 #coronavirus #coronavirusoutbreak #covid19” or tweets that make announcements about TV interviews, such as “I gave interview last month where I was asked “Is COVID-19 going to be like Zika, where nobody was really affected in the end?” and “It’s great to see the media interviewing actual experts on epidemics about #COVID19!” The distribution of the relevant tweets was as follows: actions and recommendations (2827/10,096, 28%), fighting misinformation (2019/10,096, 20%), information and knowledge (911/10,096, 9%), health care system (909/10,096, 9%), symptoms and illness (810/10,096, 8%), immunity (707/10,096, 7%), testing (605/10,096, 6%), and infection and transmission (503/10,096, 5%).

**Figure 5:**
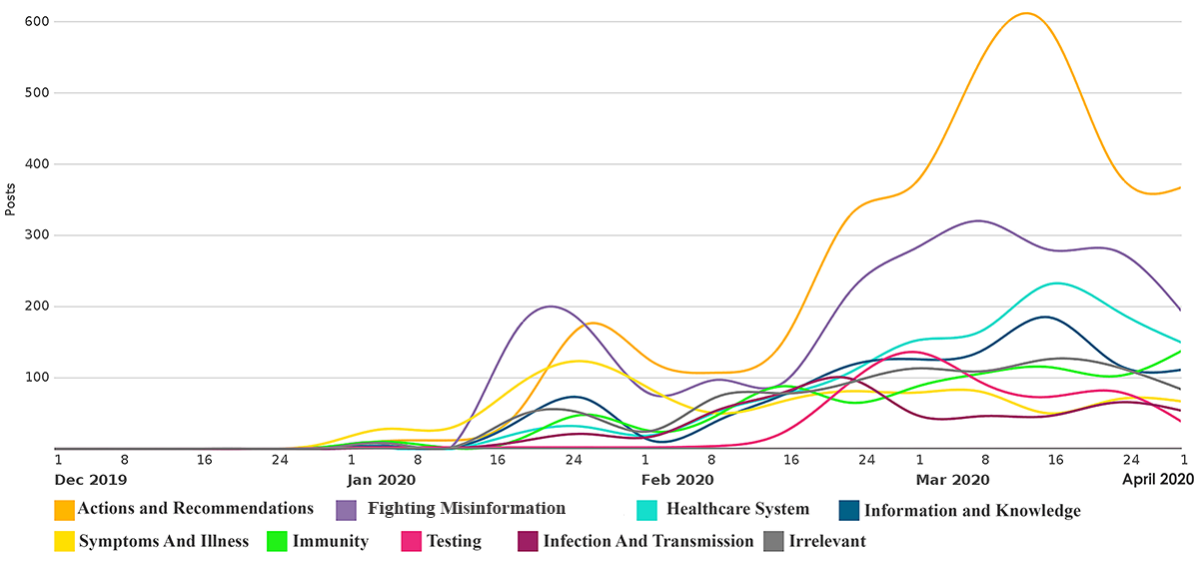
Trends of tweets per category from December 2019 to April 2020.

Figure 5 shows the volume of tweets over time by category. As shown in the figure, the number of posted tweets increased with time. More tweets were posted as the number of COVID-19 cases increased. The number of tweets posted about actions and recommendations increased noticeably, followed by tweets posted about fighting misinformation.

### Actions and Recommendations

Overall, the tweets revealed the important topics about the COVID-19 pandemic that medical professionals discussed during the period of the study. Medical professionals provided a wide range of actions and recommendations that must be considered by the government, public health officials, and individuals. These actions and recommendations mainly focused on flattening the curve, quarantine, self-isolation, social distancing, staying at home, and personal self-care. According to Multimedia Appendix 2, tweets related to “must quarantine,” “social distancing,” “flattening,” and “curve” dominated this category. Example tweet:

This is the time to #preparenotpanic for #COVID2019 Here are my tips 1. good hand hygiene (wash hands with soap and water or use alcohol-based hand gel) 2. cover your cough and sneeze 3. if sick stay at home 4. discuss sick leave rules with work.

### Fighting Misinformation

Medical professionals also discussed concerns related to misinformation and how dangerous and misleading it is to share and report such information. Furthermore, they encouraged the public to seek updates from government officials and trusted sources. According to Multimedia Appendix 3, tweets related to “misinformation” and “disinformation” dominated this category. Medical professionals discussed information related to the virus infection and transmission. Example tweet:

Along with infectious disease epidemics come misinformation/hysteria epidemics. The latter spreads faster and is just as dangerous. We need responsible reporting; accurate information; and the media needs to avoid using panic/fear to sell headlines. #coronavirus #nCoV2019

### Health Care System

Medical professionals shared their opinions regarding the health care system during the COVID-19 pandemic. They shared information and concerns regarding health care workers and their safety and whether the health care system can accommodate the increasing numbers of patients with COVID-19. According to Multimedia Appendix 4, tweets related to “healthcare,” “hospitals,” “system,” “patients,” and “workers” dominated this category. Example tweet:

We will need LOTS of help, including delivery of food and medicine, support of health care facilities, or direct patient care.

### Information and Knowledge

Medical professionals posted tweets about general COVID-19 pandemic information and knowledge. This information and knowledge included recent statistics and comparisons between countries and general information about the virus not directly related to any of the other categories. According to Multimedia Appendix 5, tweets related to “information,” “important,” and “knowledge” dominated this category. Example tweet:

#coronaviruses are enveloped viruses, meaning they are coated with a membrane derived from the host cell.

### Symptoms and Illness

Medical professionals also shared their knowledge about the symptoms and illnesses associated with COVID-19. According to the tweets in this category, people infected with COVID-19 can have a range of symptoms, from none to severe. The tweets indicated that some patients with COVID-19 can have no immediate symptoms or no symptoms at all (asymptomatic), most patients seem to have no or mild symptoms, and some patients have pneumonia or breathing issues. According to Multimedia Appendix 6, tweets related to “symptoms,” “asymptomatic,” “illness,” “mild,” and “severe” dominated this category. Example tweet:

People who have contracted new #coronavirus are showing a wide range of symptoms. Of known cases, most people exhibit milder symptoms, but about 1 in 5 people have severe illness, including #pneumonia and respiratory failure.

### Immunity

Medical professionals shared thoughts and opinions about how our immune systems respond and react to the virus as well as theories related to immunity. According to Multimedia Appendix 7, tweets related to “immunity,” “herd,” “strategy,” and “immune” dominated this category. Example tweet:

Collecting antibodies from those who recover from coronavirus infections is certainly a strategy to consider, & various countries are looking at this. Personally, I would prefer to give convalescent plasma to a patient with knowledge that it contains a decent amount of antibodies.

### Testing

The medical professionals also discussed testing in their tweets. These discussions were mainly about testing as the most viable option to control the disease, concerns about testing, and the need to scale up testing and expand testing capabilities. According to Multimedia Appendix 8, tweets related to “testing,” “test,” “kits,” “lab,” “mild,” and “severe” dominated this category. Example tweet:

We need to be thinking outside the box: drive-thru testing & home-based testing. This will help expand testing options for patients.

### Infection and Transmission

Medical professionals also discussed information related to virus infection and transmission. According to Multimedia Appendix 9, tweets related to “transmission,” “spread,” “outbreak,” and “infected” dominated this category. Example tweet:

Its good news that young children appear not to suffer severe #COVID19 illness. Unfortunately, the bad news is that these kids can readily spread the #coronavirus to others who are at much higher risk for serious illness.

## Discussion

### Principal Findings

The collected data and analysis show that social media content reveals important topics that medical professionals perceive as relevant to the ongoing discourse about the COVID-19 pandemic. These topics are mainly related to actions and recommendations, fighting misinformation, the health care system, information and knowledge, symptoms and illness, immunity, testing, and infection and transmission. Interestingly, tweets relating to actions and recommendations and concerns about misinformation accounted for more than 50% of relevant tweets, while health care system–related tweets accounted for less than 10% of relevant tweets. This is revealing given that discussion of shortages of medical supplies and limitations of the health care system seems to dominate mainstream media. However, while medical professionals are concerned about the health care system, from their perspective, the importance of actions and recommendations reflects a proactive stance to combat a pandemic that currently has no effective treatment and for which a vaccine will not be available for a long time. Tweets in this category peaked around mid-March, coinciding with the ongoing effort to curtail the pandemic, and included extensive references to approaches such as social distancing, quarantining, and contact tracing. These approaches are considered to be the first response to new infectious diseases [23]. While the volume of tweets has declined since mid-March, there was an uptick toward early April, potentially coinciding with conversations associated with the appropriate timing for “opening” the economy and associated measures that may be needed to keep the pandemic in check.

Furthermore, misinformation is a major concern for medical professionals; this was addressed in many tweets, which emphasized how this misleading information could lead to infection of more people with the virus. In a sense, some tweets suggested that the spread of misinformation was equally as disconcerting as the spread of COVID-19. Although the actual process by which such infection and spread could occur due to misleading information is not clear, there is ongoing effort by government and public health organizations such as the Centers for Disease Control and Prevention (CDC) and WHO to disseminate credible information about the state of the pandemic. This effort is imperative to develop interventions to fight misinformation in cases where high quality information may literally be a life-and-death concern [24]. As of April 1, 2020, this topic remained second with respect to tweet volume, indicating continued concern. Implications for public health include the need to expand the reach of credible information about various aspects of the virus, including symptoms, treatment, testing, vaccination, and progression. It is also important to increase public awareness about the duty to share information wisely and the importance of seeking information from trusted sources such as medical professionals.

Concerns were also shared about health care systems and health care workers. Medical professionals expressed concern that the increase in the number of cases will lead to collapse of the health care system and that the shortage of medical personal protective equipment (PPE) will increase the likelihood that health care workers will be infected. According to the CDC [25], it is critical to make every effort to protect the essential national workforce of health care providers, both at work and in the community. Also, CDC data show that PPE shortages are posing challenges to the health care system because of the COVID-19 pandemic [26]. Examples of suggested measures include scaling up existing facilities, provisioning field hospitals, and directing resources to support ailing health care infrastructure in a timely and proactive manner. Interestingly, the number of tweets showed a declining trend toward the end of the analysis period, potentially reflecting the global effort to ramp up health care infrastructure and the adaption of health care providers to the fledging health crisis.

In addition, medical professionals shared their knowledge and information about the symptoms associated with COVID-19; they stated that a person with COVID-19 can show a wide range of symptoms and it is even possible that they will show no symptoms at all. This is aligned with the existing literature, where accumulating evidence is indicating that a substantial fraction of people infected with COVID-19 are asymptomatic [27]. These posts reflect less than 20% of all relevant posts; however, they reflect another opportunity for medical professionals to influence the course of the pandemic by helping to disseminate credible and accurate information about the disease. This role can also extend to proactively debunking misinformation. While the volume of tweets showed a declining trend after mid-March, it is encouraging to note that there was an uptick toward the end of the analysis period.

Issues related to how the human immune system acts and reacts with the virus were also discussed. Most notably, medical professionals were concerned about the fact that some countries are considering herd immunity as an option to address the COVID-19 pandemic. This option is criticized because it is practically impossible to perfectly tune actual interventions without exceeding or undershooting the capacity of the health care system [28]. Interestingly, immunity shows a consistent upward trend during the analysis period among all topics. This trend is likely to continue as health care professionals, policymakers, and communities attempt to determine the role of the immune system (particularly post-infection) in suppressing the likelihood of future infections and how these findings can impact future courses of action.

Medical professionals tweeted about testing as the most urgent and efficient option to control the spread of COVID-19 while there is no effective treatment or vaccine. Given the magnitude of the COVID-19 pandemic, effective testing can reduce or prevent the need for much greater intrusions [29]. Suggested measures included increasing testing, surveillance, and detection as much as possible, adopting drive-through testing, and rapid scaleup of diagnostic testing outside of hospitals.

Finally, information about how the virus infects people, transmits from person to person, and spreads in communities was shared by different medical professionals. In this category, there was a significant emphasis on the role of the public in stemming the transmission of the infection. This information included the need to practice social distancing, basic personal hygiene, and self-quarantining after potential exposure to COVID-19.

### Limitations

This research has a number of limitations due to its reliance on social media. For example, despite the breadth of tweets collected, not all medical professionals use Twitter, and those who do use Twitter use a significant amount of discretion with respect to their level of engagement with the platform. There are also temporal and geographic dimensions [5,6] that are not necessarily captured. The findings from the analysis could be improved through additional refinement of the defined categories and by focusing on specific categories (eg, actions and recommendations). In addition, the data collection could be complemented with surveys of medical professionals with more focused and specific questions to better understand their specific concerns and experience.

### Conclusions

In this research, we analyzed tweets by medical professionals on social media to understand topics, insights, and information about the COVID-19 pandemic outbreak. Using a mixed methods approach that blended social media analytics and qualitative analysis, this research revealed trending themes and topics of concern by medical professionals about the novel coronavirus. While this health crisis is still unfolding, this study provides a unique perspective of medical professionals during the early stages of the pandemic outside of China. At this stage, a sizeable volume of tweets pertained to proactive actions to combat the virus and to recognition of the scale of the spread of misinformation as well as its adverse effects on the ongoing effort to fight the pandemic. Other issues characterizing this stage included concern about the current status of the health care system, the dissemination of information about the disease, the role of testing to better assess the scope of the crisis and properly target mitigation efforts, and the potential response of the human immune system.

## Appendix

Multimedia Appendix 1 Hierarchy Chart from Automatic Coding and Codebook for Labeling Categories.

Multimedia Appendix 2 Topics of tweets in the Actions and Recommendations category.

Multimedia Appendix 3 Topics of tweets in the Fighting Misinformation category.

Multimedia Appendix 4 Topics of tweets in the Health Care System category.

Multimedia Appendix 5 Topics of tweets in the Information and Knowledge category.

Multimedia Appendix 6 Topics of tweets in the Symptoms and Illness category.

Multimedia Appendix 7 Topics of tweets in the Immunity category.

Multimedia Appendix 8 Topics of tweets in the Testing category.

Multimedia Appendix 9 Topics of tweets in the Infection and Transmission category.

## Abbreviations

CDC: Centers for Disease Control and Prevention
COVID-19: coronavirus disease
PPE: personal protective equipment
WHO: World Health Organization
