# Supplementary material for: Mining Physicians’ Opinions on Social Media to Obtain Insights Into COVID-19: Mixed Methods Analysis
Source: JMIR Public Health Surveill. 2020 Jun 18;6(2):e19276. doi: 10.2196/19276 (PMC7304257; doi:10.2196/19276)
Supplement: Multimedia Appendix 1 [file publichealth_v6i2e19276_app1.doc]

**Multimedia Appendix**

**Hierarchy Chart from Automatic Coding.**


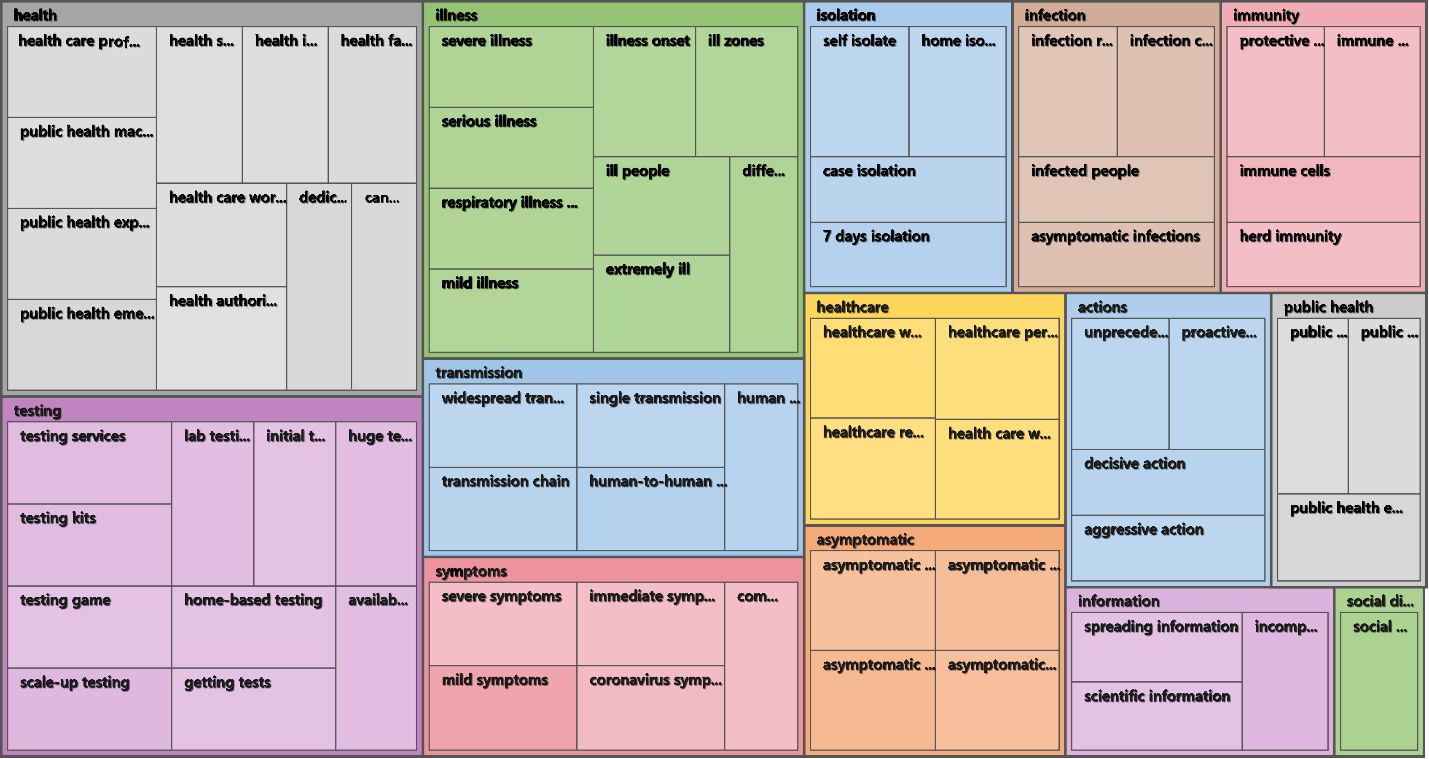


**Codebook for Labeling Categories**

| Category | Description | Keywords | Example |
| --- | --- | --- | --- |
| Actions and Recommendations | Describe medical professionals’ thoughts and opinions about proactive actions and policies needed to control the outbreak as well as recommendations for the public. | Actions, Quarantine, Self-isolation, Social Distancing, Hygiene, and Sanitizer | *“If you test + for #Covid19 you should be isolated while contagious. If you’re exposed to #Covid-19 but not yet tested positive you should self-quarantine for 14 days. This can be done safely at home if you’re not too sick and need care.” “The key policy recommendations to come from this analysis of how Wuhan eventually controlled #COVID19 - heavy duty quarantines and movement restrictions - are the very things not being done in the US.”, “Now it’s time to do our duties, which, frankly, are not that tasking: wash hands, stay at home so that, you know what, we save ourselves & the rest of the world #COVID19”, and “It's high time we practice social distancing measures. - cancel in person seminars, meetings, conferences. - enable remote conferencing. - stop traveling & stay home. - protect the vulnerable (elderly, immunocompromised). - stop handshakes, high fives or hugs. #COVID19”,* |
| Fighting Misinformation | Describe medical professionals’ thoughts and opinions about misinformation and fake news and how it relates the coronavirus outbreak. | Warning from incomplete Information, Rumors, Misinformation, Fake News, and Disinformation. | *“The last 9 weeks of #COVID19 response was abysmal. Denialism and complacency are the enemies of epidemic control. Denialism of facts is actually the worse kind of misinformation because it assumes the data isn’t even there.”, “There's not only a new virus spreading - there's misinformation about the virus that fuels its spread and hinders efforts to stop contagion”, and “Again, this is just one account out of many. Please, use trusted and verified sources when assessing the #COVID19 #coronavirus situation. It's hard for medical personnel and public planners to grasp the level of malicious misinformation that is being injected into our comms”.* |
| Healthcare System | Describe medical professionals’ thoughts and opinions about the status of the healthcare systems as well as healthcare workers during the coronavirus outbreak. | Emergency Room, Public Health Experts, Healthcare System, Health Authorities, Healthcare workers, and Health facilities | *“Very important that we improve turn-around time for ruling out healthcare workers for #COVID19 so that we don’t lose large portions of our healthcare workforce”, “Asking our brave healthcare providers to care for #Covid_19 patients without personal protective equipment is like marching soldiers into war without helmets or flak jackets. We’ve GOT to get them the gear they need to protect themselves and their families - and the rest of us”, “This was the right thing to do. What we need is time. Time to build up our healthcare capacity”, “What next? Lots of things. One of the most important among them is acting rapidly to increase healthcare capacity”, “Our nurses, doctors, hospital staff and laboratorians continue to show up to work - where chances of infection w #COVID19 are highest”, and “Healthcare workers in NYC are wearing surgical masks instead of respirators because that's all we have. Please donate your stash of N95 masks to your nearest hospital”* |
| Information and Knowledge | Describe general information and knowledge about coronavirus. | Information, support, please, think, thought. | *“The other part of this is also what a doubling of numbers means when you’re later in the epidemic as well #COVID19. At a very basic level, the difference between going from 25 to 50 cases over a set period of time (doubling time) is very different than when 500 becomes 1000 etc”, “#covid19 is a serious global public health challenge and Canadians are rightly concerned”, “Outbreak and disease is called #covid19 & virus is named sars-cov-2”, and “coronaviruses can retain infectivity on steel/plastic type surfaces for hours at 30'C and 80% humidity”.* |
| Symptoms and Illness | Describe medical professionals’ thoughts and opinions about different kinds of coronavirus symptoms and related illness. | Illness onset, Mild Illness, Respiratory Illness, Severe illness, Common Symptoms, Immediate symptoms, Mild symptoms, Severe symptoms, and Pneumonia. | *“Interesting report on presenting symptoms of #nCoV2019 which don’t just include #fever #cough but also #neurological #cardiovascular #ophthalmic and #myalgias.” and “First symptoms of COVID in most cases are dry cough and fever, not a runny nose!”* |
| Immunity | Describe medical professionals’ thoughts and opinions about how our immune responses and reacts to the virus as well as theories related to immunity. | Immunity, Herd Immunity, Immune Cells, Antibodies, and Protective Immunity. | *“Presently, our platform is the world’s best for antibody discovery. The platform can take immune cells from essentially any source with a natural immune system and isolate the B cells that make antibodies with the therapeutic properties of interest. #COVID19”, “I am deeply uncomfortable with the message that UK is actively pursuing ‘herd immunity’ as the main COVID-19 strategy”, “This new #coronavirus likely will become like another seasonal flu virus. Although you become immune to this particular strain, over time, it will mutate. You will only be partially immune and can get infected, but the illness is likely less severe because of partial immunity”, “The communication about COVID science has generally been clear in the UK, but talk of ‘herd immunity as the aim’ is totally wide of the mark. Having large numbers infected isn’t the aim here, even if it may be the outcome”, and “In a single day of screening, we can screen through millions of immune cells, and find the best antibodies that will then go on to be developed as therapeutics”* |
| Testing | Describe medical professionals’ thoughts and opinions about coronavirus testing and labs. | Home-Based Testing, Scale-up Testing, Testing Kit, Testing Services, Diagnostic Labs, and Lab supplies. | *“Can’t know if there is an epidemic if we don’t test for #COVID19”, Testing is severely limited in all states. It is limited by lack of test kits. It is limited by lack of financial means #coronavirus #testvirusnow”, “Getting the academic labs stood up to do #covid19 testing will greatly expand our screening capacity”, “Regardless of how we got here, we now need to ramp testing capability quickly to avert a wider U.S. epidemic of #coronavirus”, “We need to be thinking outside the box: drive-thru testing & home-based testing. This will help expand testing options for patients”, “Coronavirus testing kits have not been widely distributed to our hospitals and public health labs.”, and “The country’s priority now is to increase testing and encourage citizens to protect themselves”.* |
| Infection and transmission | Describe medical professionals’ thoughts and opinions about how coronavirus transmit and infect people. | Human-to-human Transmission, Transmission Chain, Widespread Transmission, Asymptomatic Infections, Asymptomatic Spread, Asymptomatic Infections, and Risk. | *“Current evidence suggests that this #coronavirus can spread via fecal-oral route and may be responsible for transmission of #2019nCoV in an apartment building in #China that had faulty plumbing”, “COVID-19 is a *respiratory tract* infection, not a blood infection.”, “Person-to-person transmission has been seen in at least 3 countries outside China”, and “Hopefully the public health machine can contain this before the virus figures out efficient human to human transmission. Not a good race to be in”* |
| Irrelevant | The post that has no related content for any of the above categories |  | *It’s great to see the media interviewing actual experts on epidemics about #COVID19!i'll be on tv at ~7:40 pm eastern. #2019ncov @coronavirus #coronavirusoutbreak* |
