# Supplementary figures and images for: Mining Physicians’ Opinions on Social Media to Obtain Insights Into COVID-19: Mixed Methods Analysis
Source: JMIR Public Health Surveill. 2020 Jun 18;6(2):e19276. doi: 10.2196/19276 (PMC7304257; doi:10.2196/19276)

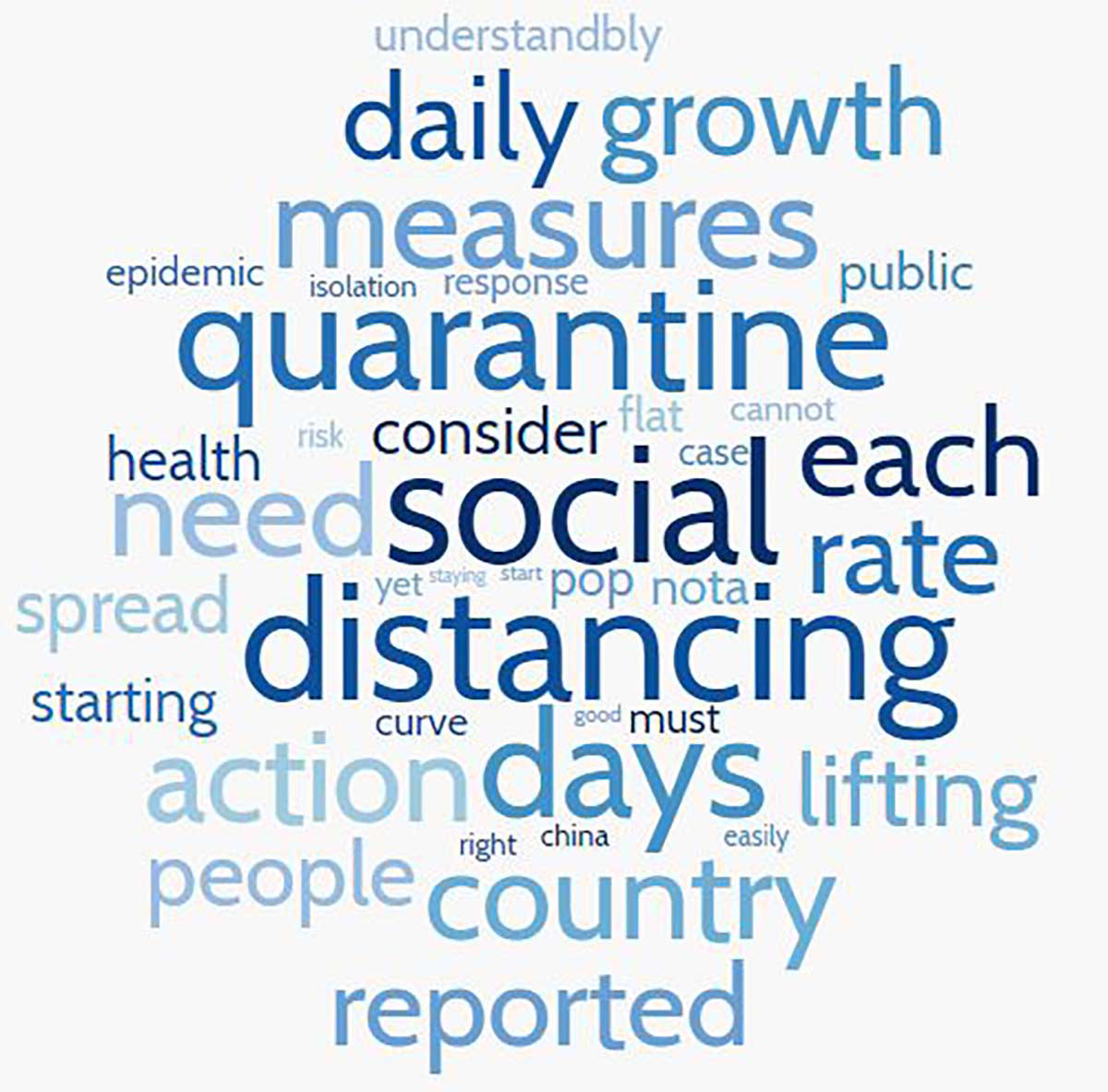

Supplement: Multimedia Appendix 2 [file publichealth_v6i2e19276_app2.png]

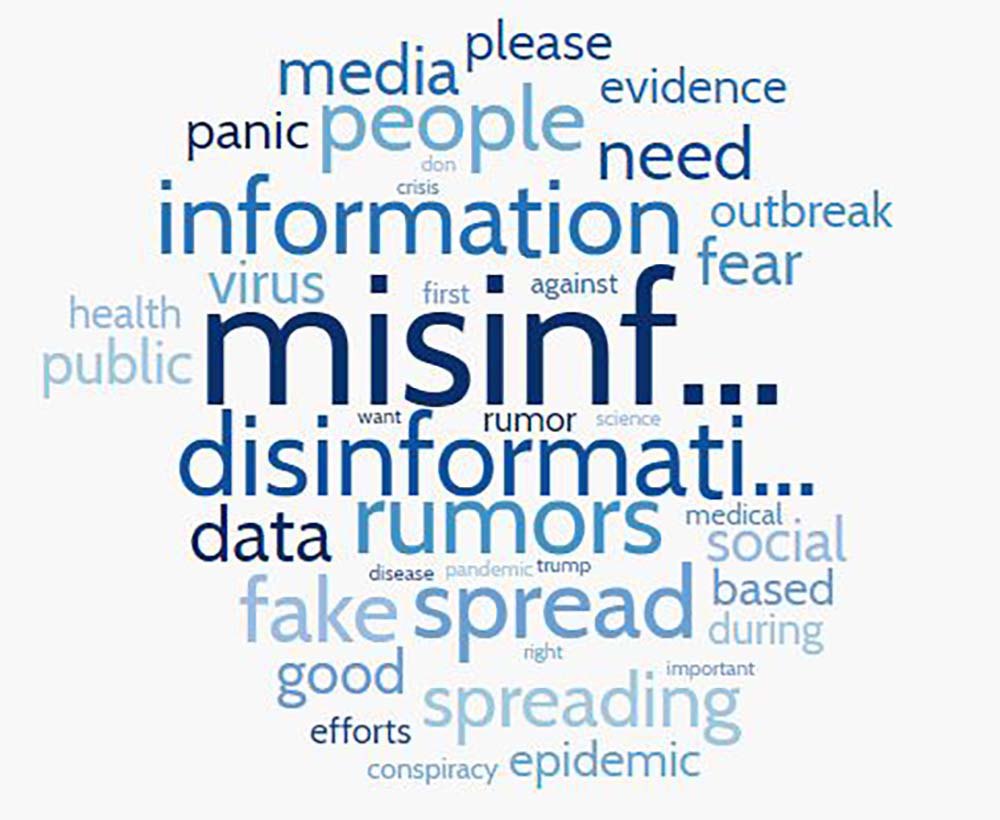

Supplement: Multimedia Appendix 3 [file publichealth_v6i2e19276_app3.png]

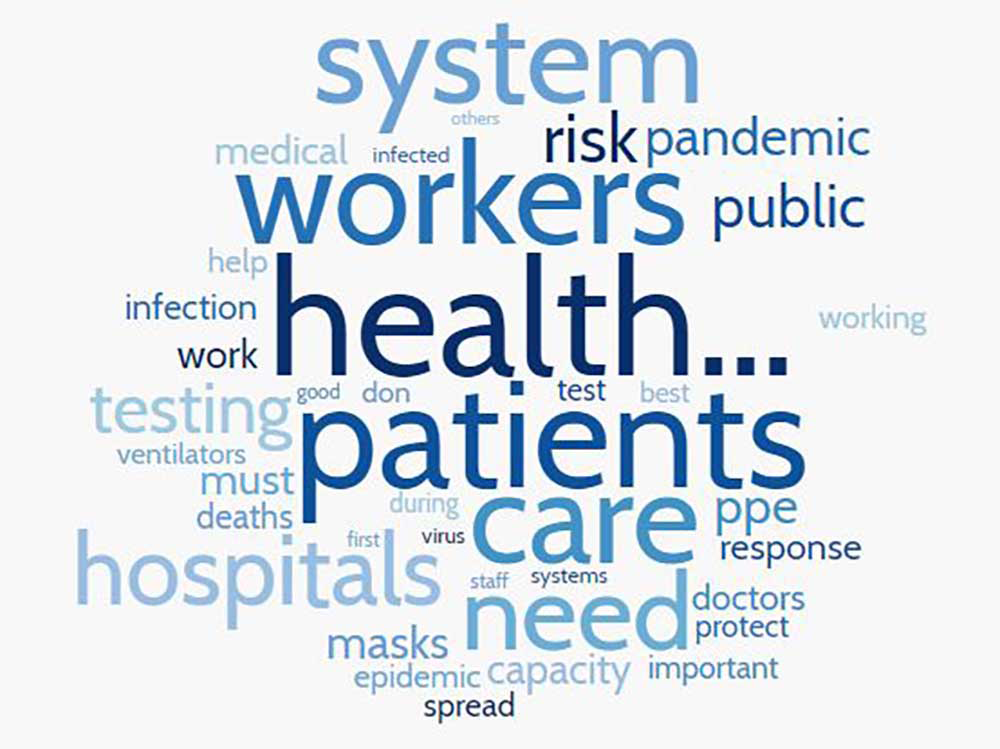

Supplement: Multimedia Appendix 4 [file publichealth_v6i2e19276_app4.png]

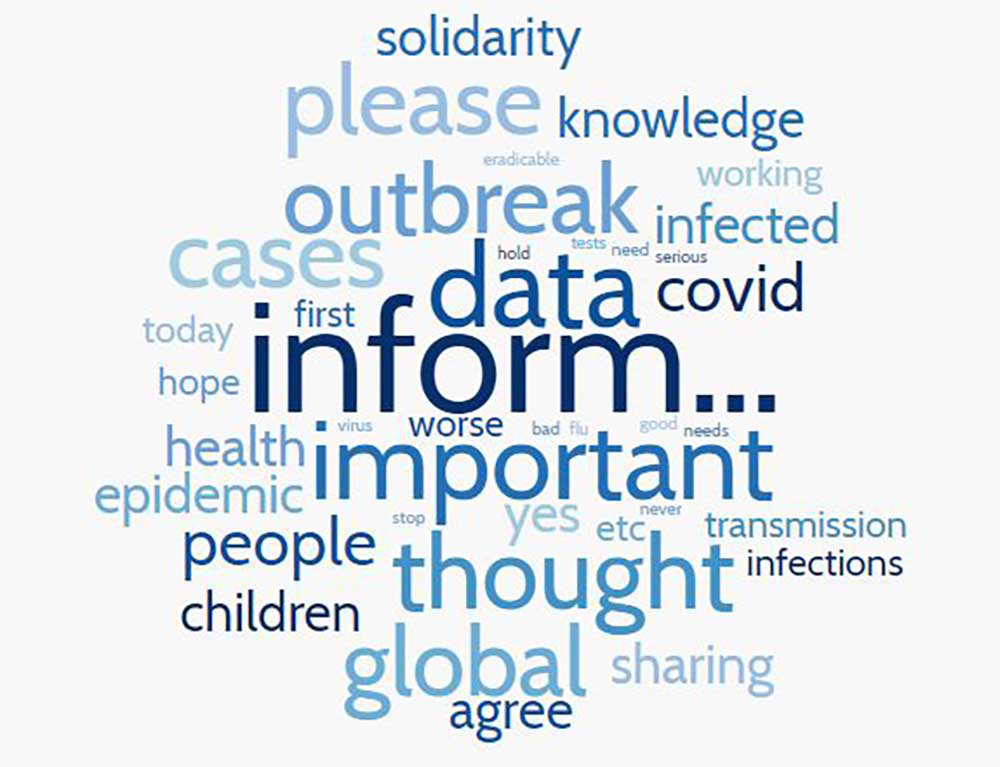

Supplement: Multimedia Appendix 5 [file publichealth_v6i2e19276_app5.png]

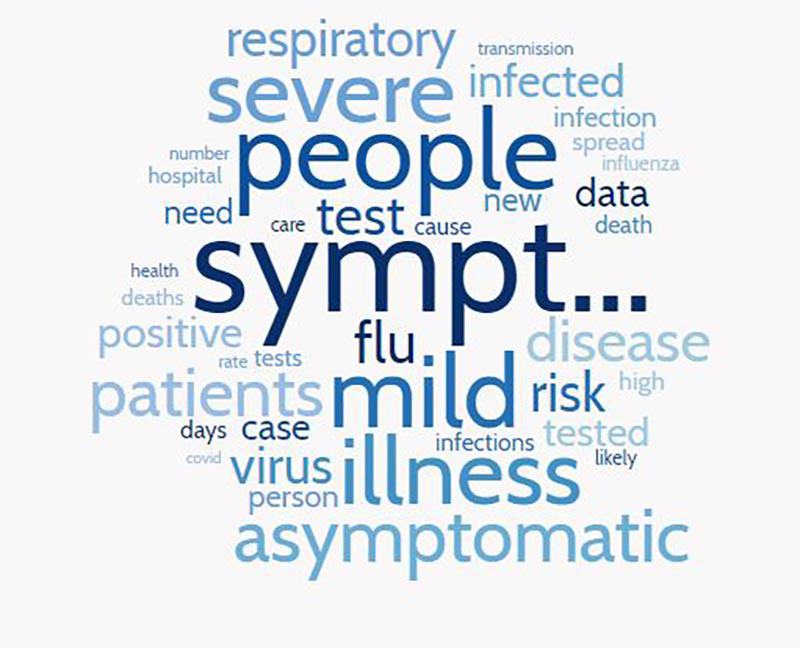

Supplement: Multimedia Appendix 6 [file publichealth_v6i2e19276_app6.png]

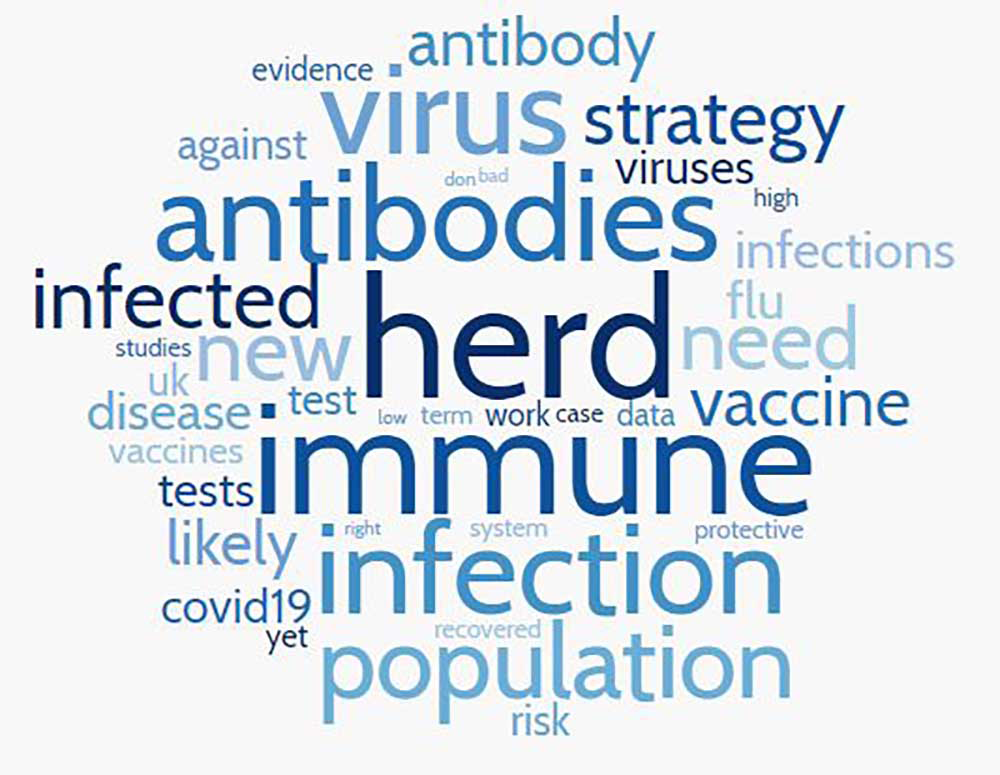

Supplement: Multimedia Appendix 7 [file publichealth_v6i2e19276_app7.png]

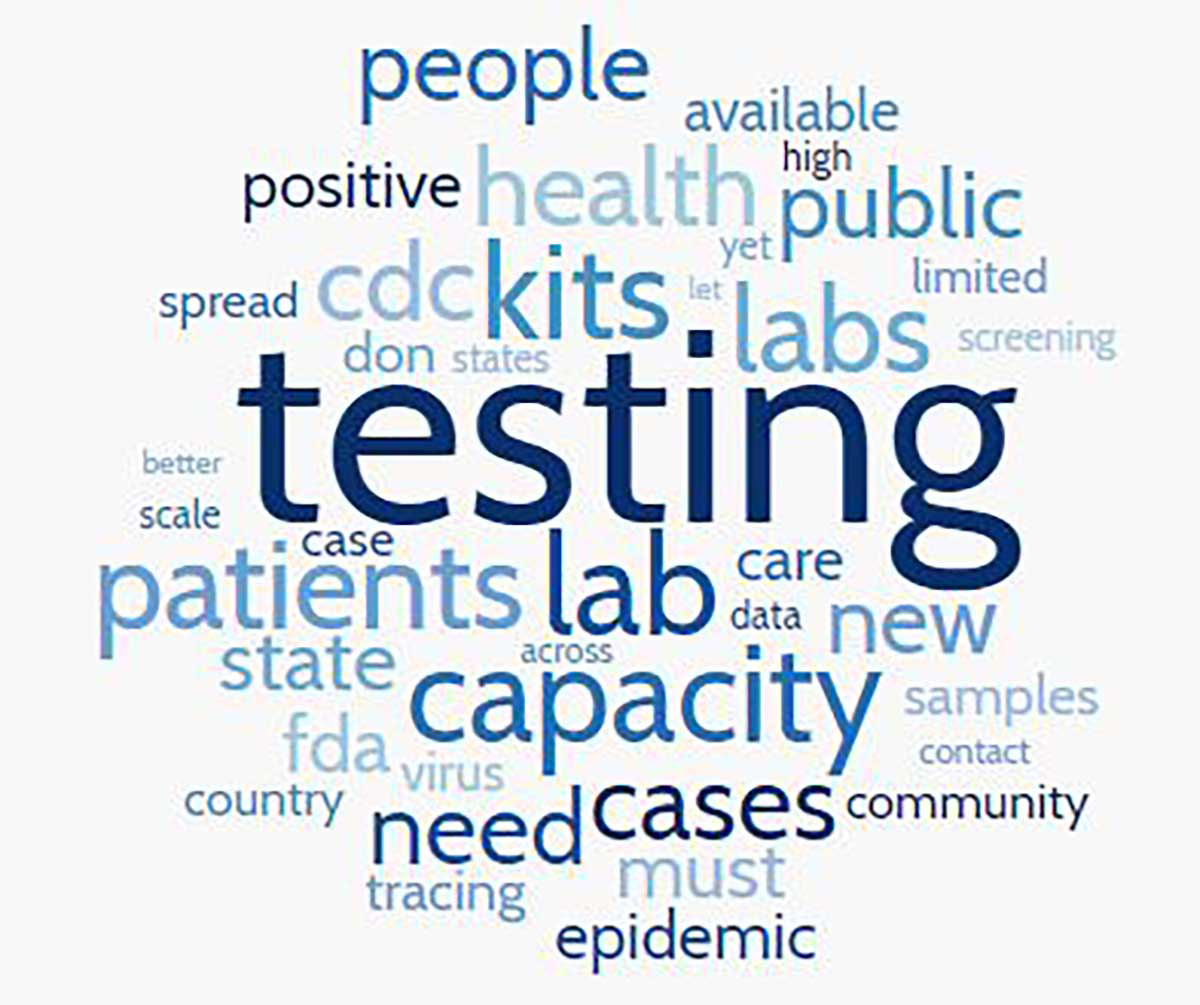

Supplement: Multimedia Appendix 8 [file publichealth_v6i2e19276_app8.png]

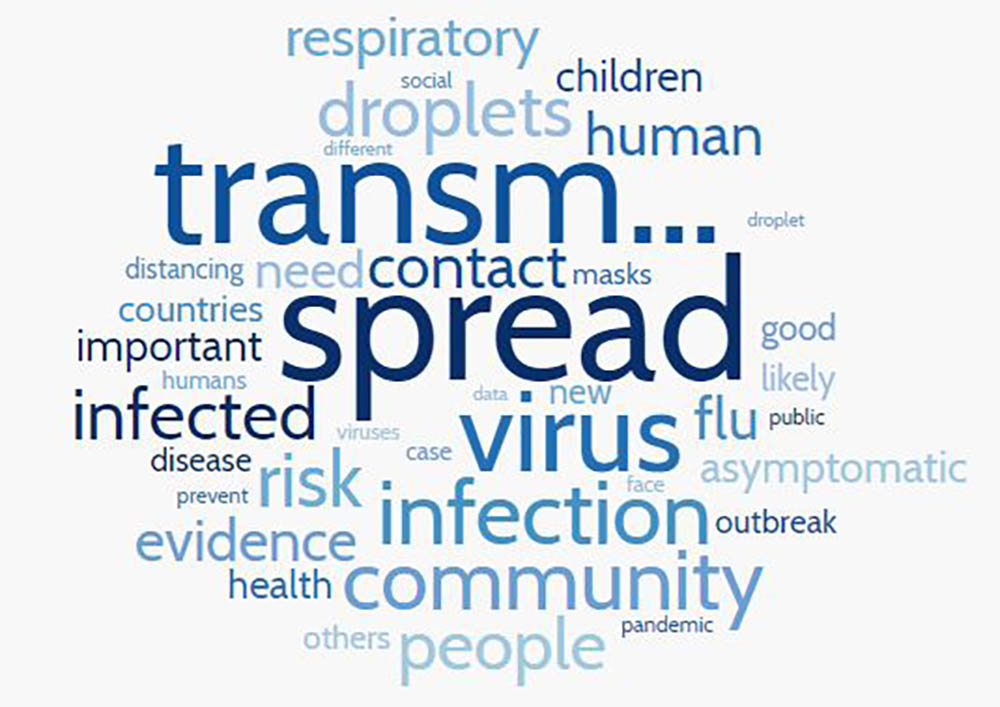

Supplement: Multimedia Appendix 9 [file publichealth_v6i2e19276_app9.png]
